# Diagnostic value of FDG PET-CT in differentiating lung adenocarcinoma from squamous cell carcinoma

**DOI:** 10.1186/s41824-024-00187-8

**Published:** 2024-01-30

**Authors:** Adem Maman, Sadık Çiğdem, İdris Kaya, Rabia Demirtaş, Onur Ceylan, Sevilay Özmen

**Affiliations:** 1 Department of Nuclear Medicine, Faculty of Medicine, Atatürk University, Erzurum, Turkey; 2 Vocational School of Health Services, Istanbul Aydın University, Istanbul, Turkey; 3 Department of Radiology, Private Buhara Hospital, Erzurum, Turkey; 4 Department of Medical Pathology, Faculty of Medicine, Atatürk University, Erzurum, Turkey

**Keywords:** Non-small cell lung cancer, Squamous cell carcinoma, Adenocarcinoma, Fluorine-18 fluorodeoxyglucose positron emission tomography

## Abstract

**Background:**

Lung cancer is the leading cause of cancer-related deaths worldwide. The combination of fluorine-18 fluorodeoxyglucose positron emission tomography (18F-FDG PET) and computed tomography (CT) has a major impact on the diagnosis, staging, treatment planning and follow-up of lung cancer patients. The maximum standardized uptake value (SUVmax) is an easily performed and most widely used semi-quantitative index for the analysis of FDG PET images and estimation of metabolic activity. This study aimed to investigate the role of PET/CT in differentiating adenocarcinoma (ADC), the most common lung cancer, from squamous cell carcinoma (SCC) by comparing FDG uptake measured as SUVmax.

**Results:**

Between 2019 and 2022, 76 patients diagnosed with non-small cell lung cancer (NSCLC) at the Department of Pathology, Atatürk University Faculty of Medicine, with histopathologic evidence of adenocarcinoma or squamous cell carcinoma, underwent retrospective analysis using PET/CT scanning to measure PET parameters of the lesions and compare them with histopathology. Among 76 NSCLC patients included in the study, 43 (57%) were histopathologically diagnosed as ADC and 33 (43%) as SCC. SUVmax, SUVmean, metabolic tumor volume (MTV) and total lesion glycolysis (TLG) values of lesions in patients with SCC were statistically significantly higher than those in patients with ADC (*p* values 0.007, 0.009, 0.003 and 0.04, respectively).

**Conclusions:**

Lung SCC has higher metabolic uptake values than ADC, and PET/CT can be used to differentiate them.

## Introduction

Lung cancer is the most common cancer worldwide and is responsible for most cancer-related deaths. According to the World Health Organization (WHO), lung cancer is the leading cause of cancer deaths globally (Mattiuzzi and Lippi 2019). Lung cancers are divided into two main groups: small cell lung cancer (SCLC) and non-small cell lung cancer (NSCLC). NSCLC is the most common type and accounts for approximately 80–85% of all lung cancer cases. The main histologic types of NSCLC are adenocarcinoma (ADC) and squamous cell carcinoma (SCC) (Goldstraw et al. 2007). The location within the lung is one of the differences between SCC and ADC. SCC tends to be central and may have internal cavitation, while ADC tends to be peripheral and solid (Gharraf et al. 2020). Lung adenocarcinoma is the most common type of lung cancer, accounting for about 40% of all lung cancers. It tends to arise in the periphery of the lungs as it originates from small airway epithelial cells that secrete mucus and other substances (Zappa and Mousa 2016). SCC is the second most common type of lung cancer after lung adenocarcinoma and originates from the bronchial surface epithelium. Tumor cells are characterized by a squamous appearance similar to that observed in epidermal cells. Lung SCC is more strongly linked to tobacco smoking than other forms of NSCLC. It usually arises in the center of the larger bronchi and often metastasizes to the locoregional lymph nodes early in its course, but spreads outside the thorax later than other major types of lung cancer (Sabbula et al. 2023). Early and accurate diagnosis of lung cancer is invaluable for successful treatment and good outcomes. The 5-year survival rate of small, localized stage 1 NSCLCs with surgical resection is approximately 70–90%. However, the majority of patients, 3 out of 4, are diagnosed at an advanced stage and have a poor prognosis (Goldstraw et al. 2007; Walters et al. 2013).

Positron emission tomography/computed tomography (PET/CT) with fluorodeoxyglucose (FDG) labeled with fluorine-18 (F-18), a glucose analog, is now routinely used in clinical practice for both the evaluation of lung nodules and the staging of lung cancer. Due to its high diagnostic accuracy in lung cancer, FDG PET/CT imaging is now included as a routine diagnostic modality in many clinical lung cancer guidelines (Ettinger et al. 2019; Kitajima et al. 2016). FDG PET/CT has become an important tool in the diagnosis and staging of NSCLC. The maximum standard uptake value (SUVmax) in FDG PET/CT is the ratio of activity in tissue per unit volume relative to the dose carried by body weight. SUVmax of primary tumors has been found to correlate with stage, nodal status, histological type, differentiation and tumor progression in patients with NSCLC. Furthermore, high SUVmax value has been shown to have serious effects on prognostic factor in patients with NSCLC (Shimizu et al. 2014). However, the application of treatments other than surgery in patients with FDG PET/CT positive lesions requires histopathologic confirmation (Ettinger et al. 2019). There is a 20% risk of pneumothorax during transthoracic biopsy. With FDG PET/CT imaging, it may be possible to overcome this risk in many patients with lung nodules. In addition, in the case of a malignant lesion, a possible pneumothorax may delay other procedures for diagnostic evaluation, which increases the importance of non-invasive FDG PET/CT imaging (Madsen et al. 2016).

In this study, we aimed to investigate whether semi-quantitative parameters of FDG PET/CT correlated with the main histological types of NSCLC, ADC and SCC, in retrospectively screened NSCLC patients. In particular, we tried to analyze whether SUVmax, SUVmean, metabolic tumor volume (MTV) and total lesion glycolysis (TLG) values of FDG PET/CT differed in patients diagnosed with lung SCC and ADC.

## Materials and methods

### Study design

Seventy-six NSCLC cases diagnosed as adenocarcinoma and squamous cell carcinoma with pneumonectomy, lobectomy, segmentectomy and wedge resection materials in the pathology department of the university hospital, between 2019 and 2022, were included in this study. Information on age, gender, tumor histological type, tumor diameter, lymph node involvement, visceral pleural invasion status and pathological stage were obtained from the hospital automation system and the Ministry of Health e-Nabız system. H&E stained preparations of the resection materials were re-examined by two observers, and histopathologic typing was performed according to WHO 2021 criteria.

### PET/CT

Patients enrolled in the study were fasted for at least 6 h. Blood glucose levels were confirmed to be less than or equal to 140 mg/dl before 18F FDG infusion. 5.5 MBq/kg 18F FDG was administered intravenously 1 h before imaging. One hour after the injection, imaging was performed from the upper thigh to the head with the patient in the supine position for 3 min per bed by Biograph 6 PET/CT (Siemens Medical Systems, Germany). Whole-body PET/CT images were evaluated by an experienced nuclear medicine specialist. Clinical staging of the patients was performed according to the TNM system of the 8th version of the American Joint Committee on Cancer/Union for International Cancer Control (AJCC/UICC). Pathologic lesion areas were drawn by the nuclear medicine specialist, and Suvmax, Suvmean, MTV and TLG values were automatically measured and recorded by Siemens VIA program for FDG uptake.

### Statistical analysis

Number, percentage, mean and standard deviation were used in the evaluation of the data. Independent samples t test was used in the evaluation of normally distributed data. Mann–Whitney U and Kruskall–Wallis tests were used for data that did not show homogeneous distribution. *p* values of 0.05 and below were accepted as significant.

## Results

A total of 76 patients, 15 (19.7%) females and 61 (80.3%) males, were included in our study. The mean age of the patients was 59 ± 9.91 years (min 26, max 78). The clinical characteristics of the patients are listed in Table 1. Excluding the incidence of ADC and SCC in women, which may be explained by their regional non-smoking habits (Cornfield et al. 2009), the parameters in Table 1 show an almost homogeneous distribution between ADC and SCC. The slides of all cases were re-evaluated by two pathologists and 43 were confirmed as ADC and 33 as SCC. In addition, tumor size, pleural invasion and tumor spread within the air spaces (STAS), which affect lung cancer stage and prognosis, were evaluated. Pleural invasion was present in 22% of our cases (17 cases), while 78% (59 cases) were negative. 41 patients had STAS, while 35 patients did not. Tumor size is an important factor in the staging of lung cancers (Rami-Porta et al. 2015). Tumor size should be given in the most accurate way because it can change the stage of the tumor. In 41 cases, the tumor size was 1–3 cm and was considered as stage 1. Twenty-seven cases were stage 2, i.e., tumor size was 3–4 cm or 1–3 cm with pleural invasion. Six cases were stage 3, and two cases were stage 4. In 14 cases, lymph node metastasis was observed, but not in 37 cases. In 25 cases there were no clear data about lymph node metastasis. Lymphovascular invasion was detected in 43 cases.

**Table 1:** Demographic characteristics of the cases

Parameters	Total number (percentage)	ADC	SCC	Significance (*p* value)
Number of patients (*n*)	76	43 (57%)	33 (43%)	NA
Age (Year, Mean ± SD)	59 ± 9.9	59 ± 10.4	60 ± 9.3	0.633
Gender (F/M)				
Female	15 (20%)	15	0	NA
Male	61 (80%)	28	33	NA
Pathologic staging (*n*)				
Stage 1	41 (54%)	25	16	NA
Stage 2	27 (36%)	14	13	NA
Stage 3	6 (7%)	2	4	NA
Stage 4	2 (3%)	2	0	NA
TNM Staging				
Stage 1	26 (34%)	18	8	NA
Stage 2	36 (48%)	18	18	NA
Stage 3	10 (13%)	5	5	NA
Stage 4	4 (5%)	2	2	NA
Lymphovascular invasion (*n*)				
Positive	43 (57%)	27	16	NA
Negative	33 (43%)	16	17	NA
STAS status				
Positive	41 (54%)	31	10	NA
Negative	35 (46%)	12	23	NA
Lymph node metastasis				
Positive	14 (18%)	5	9	NA
Negative	37 (49%)	17	20	NA
NoS	25 (33%)	21	4	NA
Pleural invasion				
Positive	17 (22%)	11	6	NA
Negative	59 (78%)	32	27	NA

ADC, adenocarcinoma; SCC, squamous cell carcinoma; NA, Not Applicable

**Fig. 1:**
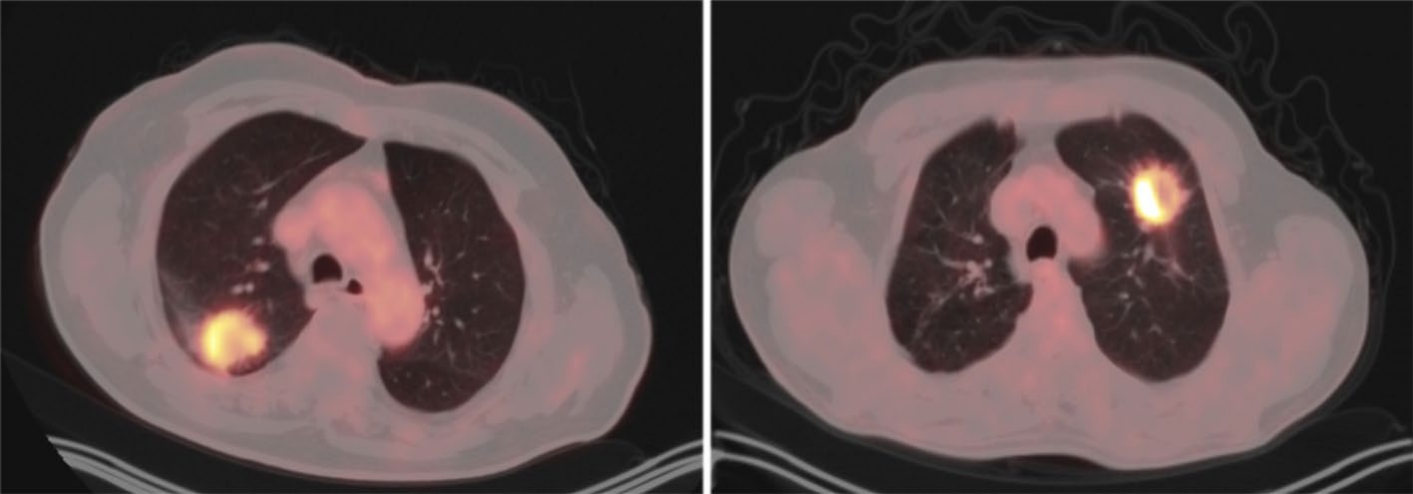
Representative pictures of ADC (left) and SCC (right). SUVmax values for ADC and SCC are 13.03 and 21.22, respectively

The values of PET parameters (SUVmax, SUVmean, MTV and TLG) are compared between ADC and SCC in Table 2. Statistical analysis was performed to assess whether there were significant differences in these variables. For the SUVmax, the mean value in ADC was 8.19 ± 6.57, whereas it was 13.18 ± 9.04 in SCC. The t test revealed a statistically significant difference between the two groups (*t* = − 2.78, *p* = 0.007), indicating that SUVmax values were significantly higher in SCC compared to ADC. Similarly, for the SUVmean, the mean value in ADC was 4.88 ± 3.74, and in SCC, it was 7.60 ± 5.06. The t test demonstrated a statistically significant difference (*t* = − 2.69, *p* = 0.009), suggesting that SUVmean values were significantly higher in SCC compared to ADC. For MTV, the mean value in ADC was 10.79 ± 21.11, and in SCC, it was 12.25 ± 14.64. The nonparametric Mann–Whitney *U* test indicated a statistically significant difference (MW-U = 430.00, *p* = 0.003), implying that MTV values were significantly higher in SCC compared to ADC. Lastly, for the TLG, the mean value in ADC was 77.81 ± 211.01, while in SCC, it was 150.47 ± 299.12. The Mann–Whitney *U* test revealed a statistically significant difference (MW-U = 522.00, *p* = 0.04), indicating that TLG values were significantly higher in SCC compared to ADC. In conclusion, the statistical analysis demonstrates significant differences in the parameters SUVmax, SUVmean, MTV, and TLG between the two groups. Representative pictures of ADC and SCC in Fig. 1 also show FDG uptake in SCC is higher than that in ADC. The results apparently suggest that there are statistically significant differences between the two groups being compared for each parameter, as indicated by the small *p* values (*p* < 0.05). SCC exhibits higher values for PET parameters compared to ADC.

**Table 2:** Comparison of PET parameters between ADC and SCC

	ADC	SCC	Test value	*p* value
SUVmax	8.19 ± 6.57	13.18 ± 9.04	*t* = − 2.78	*p* = 0.007
SUVmean	4.88 ± 3.74	7.60 ± 5.06	*t* = − 2.69	*p* = 0.009
MTV	10.79 ± 21.11	12.25 ± 14.64	MW-U = 430.00	*p* = 0.003
TLG	77.81 ± 211.01	150.47 ± 299.12	MW-U = 522.00	*p* = 0.04

## Discussion

In this study, we analyzed the variances in semi-quantitative SUVmax, SUVmean, MTV and TLG values of FDG PET/CT between lung carcinomas diagnosed with ADC and SCC. We found significant differences between ADC and SCC. According to our study, SUVmax, SUVmean, MTV and TLG values were significantly higher in SCC patients compared to ADC patients (*p* values 0.007, 0.009, 0.003 and 0.04, respectively).

Early and accurate diagnosis of lung cancer subtypes is of vital importance. Although PET/CT technology, which can perform non-invasive tumor assessment for classification, staging, efficacy and prognostic evaluation, has been shown to be useful in determining the subtype of cancers in recent studies, the parameters for its use in this field have not yet matured (Sun et al. 2023). In this study, similar to recent studies (Salem et al. 2021), we found that quantitative PET/CT values were higher in SCC (Table 2).

The advantage of FDG PET/CT is that it combines the functional-metabolic information of PET with the detailed anatomical-morphologic information of CT in a single hybrid study. PET/CT has also been used to assess metabolic activity in lung cancers. Semi-quantitative PET parameters such as MTV and TLG values have previously shown promising results in providing prognostic insight for disease progression of respiratory system tumors (Jin et al. 2018).

The mRNA and protein expression of metabolic markers has been shown to be higher in squamous cell carcinomas than in adenocarcinomas, whereas adenocarcinomas are better vascularized (Goodwin et al. 2017). Adenocarcinomas have a worse disease-free survival (DFS) compared to squamous cell carcinomas based on the potential for metastasis. Adenocarcinomas have been shown to exhibit glycolysis under normoxic conditions, whereas squamous cell carcinomas are subjected to diffusion-limited hypoxia (Schuurbiers et al. 2014). Although squamous cell carcinomas have a higher FDG uptake, which is generally considered a poor prognostic factor, adenocarcinomas have a higher metastatic potential and a worse DFS (Schuurbiers et al. 2014). These findings suggest that FDG PET should be interpreted in relation to histology. FDG PET may improve the prognostic potential of the disease and its use in histology-related treatment strategies could be expanded.

Studies on this subject are limited. Similar to the results of previous studies, Schurbiers et al. and Hyun et al. showed that SCC had a higher SUV value compared to ADC as a general trend although there was no statistically significant difference in the SUV value of static PET between ADC and SCC in their study (Schuurbiers et al. 2014; Hyun et al. 2019). This may provide a theoretical basis and technical support for early, accurate and personalized treatment. The cellular reason for this may be that SCC has a higher rate of glycolysis and less vascularization compared to the high perfusion and low rate of glucose phosphorylation in ADC (Sun et al. 2023; Vriens et al. 2012). Patnaik et al.'s meta-analysis showed that higher values of SUVmax, MTV and TLG predicted a higher risk of recurrence or death in patients with surgical NSCLC. They recommended the use of FDG PET/CT to select patients at high risk of disease recurrence or death who may benefit from aggressive therapies (Patnaik et al. 2016).

The number of patients in the study is limited for a comprehensive conclusion; nevertheless, it provides important clues for a clear distinction between ADC and SCC. Moreover, our study was performed on operated patients, so the number of stage 4 patients is small, and a more comprehensive study can be performed with a similar number of patients at each stage.

## Conclusions

In conclusion, 18F-FDG PET/CT imaging has a marked potential for the classification of NSCLC and differential diagnosis of subtypes and thus may help clinicians to improve the histopathologic diagnosis of lung cancer in a noninvasive manner.

## Data Availability

Data supporting our findings are available from the corresponding author upon reasonable request.

## Abbreviations

FDG PET: Fluorine-18 fluorodeoxyglucose positron emission tomography
SUVmax: Maximum standardized uptake value
SUVmean: Mean standardized uptake value
ADC: Adenocarcinoma
SCC: Squamous cell carcinoma
NSCLC: Non-small cell lung cancer
MTV: Metabolic tumor volume
TLG: Total lesion glycolysis
